# Congenital Malformations

**Published:** 1962-04

**Authors:** Beryl D. Corner

**Affiliations:** Consultant Paediatrician, United Bristol Hospitals and Southmead Hospital


### CONGENITAL MALFORMATIONS

### Clinical Considerations*

BY

BERYL D. CORNER, M.D., F.R.C.P.

*Consultant Paediatrician, United Bristol Hospitals and Southmead Hospital*

In this country today if a child survives the first 4 weeks after birth he has a very good chance of 70 years of life, but to attain this he still has to negotiate the hazardous perinatal period.

The perinatal mortality survey for England, Scotland and Wales showed that the highest single cause of first week deaths is congenital malformation, constituting nearly 25 per cent of deaths in March 1958. (Butler, 1961.)

Medicine is constantly providing us with new challenges, and this one must be obvious, for in addition to some of these lives which might be saved by better diagnosis and treatment, there are many more handicapped survivors. The term "congenital malformation" implies a condition which is present at birth but which is not due to the birth process; for malformation the terms anomaly, defect or deformity may be substituted. This definition covers a wide range and as well as disorders of structure, includes those of function, e.g. the congenital enzyme defects which cause widespread damage to cell chemistry with the growth of the child: phenylketonuria, galactosaemia and several similar disorders, and the congenital defects of the red cells and haemoglobin which are responsible for the congenital haemolytic anaemias.

The diagnosis of congenital malformations may be obvious at birth, or by careful examination within the first few months of infancy. Some only reveal themselves much later in life, e.g. those of the genito-urinary tract, and others only in the post-mortem room having never caused any apparent trouble. If effective treatment is to be applied, however, to all who need it, the aim should be to discover as many as possible as early as possible, The field of discovery is rich for all who seek, and it is noteworthy that with the greatly improved paediatric medical supervision of all our babies in Southmead and Bristol Maternity Hospitals how many more abnormalities, and particularly those of the heart and the nervous system are being detected at an early age.

For efficiency and economy of time and effort the search must be thorough and appropriately timed. The infant should always be examined naked and lying flat on a table or some similar surface. It is impossible to make an efficient examination when he is clothed in a gown usually three times too big and lying in the bottom of a canvas sling cot, or on the midwife's or mother's lap. The doctor's most important instrument is his eyes and thorough general inspection is the essence of the examination, and includes the eyes, the inside of the mouth and the external genitalia. Detection of the imperforate anus may require close inspection, and is occasionally missed until attempts are made unsuccessfully to insert a thermometer. The examination of any baby requires removal of the napkin, and failure to do so may conceal such an important condition as pseudohermaphroditism, which when accompanied by the suprarenal

* A paper read to the Bristol Medico-Chirurgical Society.

salt-losing syndrome is an important cause of severe failure to thrive and vomiting in early infancy.

The head should be measured and the fontanelles palpated, since this is an essential base-line for assessment of skull and brain growth. Observation of the resting posture, movements and general behaviour should be made and followed by examination of the heart, lungs and abdomen. Congenital dysplasia of the hip joint may be detected by the Ortolani method, i.e. with the baby lying supine on a table, the examiner grasps both thighs, flexes the hips to a right angle and fully abducts them. A click detected as the head of the femur slips over the rim of the acetabulum suggests dysplasia, and it is particularly important to examine all girls in this way.

When should the baby be examined? Gross external lesions are detected by the first rapid examination carried out shortly after birth, but a fuller and more careful examination should be performed on the second day. Some of the alimentary tract obstructive lesions are then manifesting themselves, but internal genito-urinary anomalies and

TABLE I

INCIDENCE OF MALFORMATIONS

| Total Births 1960 and 1961 SOUTHMEAD HOSPITAL | 8059 |
|---|---|
| Cutaneous Tissues | 22 |
| Alimentary Tract | 36 |
| Skeletal | 39 |
| Nervous System | 61 |
| Genito-Urinary | 16 |
| Cardiovascular | 42 |
| Respiratory | 2 |
| Multiple Abnormalities | 18 |
| (Stillborn | 39) |
| TOTAL | 236 |

Incidence: 2·9 per cent of ALL BIRTHS

non-cyanotic congenital cardiac lesions are rarely detectable. Many babies develop a transitory cardiac murmur during the first few hours, but most of the organic murmurs are heard later in the neonatal period, the common time being between the 10th–28th days. The third examination should be carried out at the 10th–14th day with the specific objects of noting the cardiac condition, not forgetting the femoral pulses, and the nervous system. Any baby making poor progress requires examination of stools and urine for the possibility of disease due to congenital enzyme defects. Phenylketones are not usually excreted in sufficient quantity to be detected in the urine by the "phenistix" or ferric chloride tests before 3–4 weeks in cases of phenylketonuria, therefore if routine testing is carried out for this disorder, 5–6 weeks is the best time. In two cases born in Bristol Maternity Hospital where daily urine tests were made from birth, urine chromatography revealed excess phenylalanine by the eighth day, but the ferric chloride test was negative until 24 days.

For the baby who presents no symptoms, a fourth routine examination at 3 months is the most profitable time to detect those lesions which are still hidden. Many of the early functional cardiac murmurs have disappeared and the murmurs due to organic disease have by then appeared. A few cases of non-cyanotic heart disease develop cardiac failure at 3–6 weeks which is often mistaken for respiratory infection, but the remainder should be detectable by 3 months. This is also a fruitful time for assessment of the central nervous system. Vision and hearing, muscle tone and the development of normal postures and locomotion can all be noted. If these routine examinations have been efficiently carried out it is unlikely that in the term infant any further abnormalities will be revealed in this way. In the premature infant, however, it is worth doing further routine examinations at 6 and 12 months, owing to the much higher incidence of abnormality and the later maturation.

Table I shows the incidence of all congenital malformations detected in infants born in Southmead Hospital during 1960–1961, whilst the infants were still in hospital. In 8059 births the incidence of abnormality was 2·9 per cent. This is of course a minimal incidence since some of the infants left hospital very early and nearly all by the 10th day. In a series of 3151 consecutive premature live births in Bristol from 1949 –1955 inclusive, the incidence of congenital abnormality was 8·1 per cent indicating the much greater risk to the low birth weight child. The system distribution of abnormalities shows a much greater incidence of nervous system disorder, 0·75 per cent, than elsewhere, cardiovascular anomalies were 0·5 per cent and alimentary tract and skeletal 0·4 per cent each of all births. Defects in other systems were by comparison insignificant. In the premature series quoted the distribution is different, anomalies of the cutaneous tissues e.g. all varieties of naevi, and alimentary tract each have an incidence of 1·7 per cent; nervous system and skeletal disorders were 1 per cent of all live premature births.

### TREATMENT

The aims of treatment are: firstly to save life largely by reducing perinatal mortality. There are many cases, particularly of cardiovascular anomalies, where although the child remains fairly well for some years, the disorder ultimately shortens life, so that treatment is aimed at giving the individual a longer life span. Thirdly, there are large numbers of children for whom there is no complete cure, but who can be made much happier, more comfortable, and able to live in a normal home and community life by appropriate treatment.

Treatment is often considered to be mainly surgical, but in many conditions medical care alone is possible. In either case, and particularly for the more serious malformations emphasis must be placed on team-work. A whole band of players is needed which includes all varieties of surgeons, therapists of all types, special and ordinary school teachers, social workers, psychologists, nurses, the family doctor, the paediatrician and the parents. The paediatrician must often assume the rôle of conductor, since with his special knowledge of childhood problems he must know every note of the symphony, and the parts to be played by all players so that each can be brought in at the appropriate moment, and if there is any conflict between them he must know which tune should temporarily be played loudest and which be damped down. The family doctor, with his knowledge of the home, has a special rôle to play which is to support the patient's interest at all times and to maintain the confidence of the family in the whole band's efforts.

It is of paramount importance that the first information to the parents is wisely given. "A word fitly spoken is like apples of gold in pictures of silver" applies to this perhaps more than any other medical problem, and the wrong word at this time may wreck the whole symphony; fears and pessimism implanted early are extremely difficult to eradicate and may affect the whole attitude to and treatment of the patient.

### ALIMENTARY TRACT ANOMALIES

The treatment of the more common groups of abnormality will now be briefly considered. Table II shows a detailed analysis of alimentary tract anomalies. Those which it is essential for life to recognize as quickly as possible after birth are micrognathos, as the infant may asphyxiate if the small tongue is allowed to fall back into the throat: oesophageal atresia, which we like to diagnose within the first hour by the presence of frothy mucus like foam on the sea, in the nose and mouth, the obstruction being easily confirmed by passage of a mucus catheter; exomphalos, which requires very urgent surgery if infection of the thin covering membrane and general peritonitis is to be avoided.

Anomalies of the jaws and palate are those most commonly seen and are now extremely well corrected by the plastic surgery team, although most of these babies require very careful preparation for operation to ensure satisfactory nutrition and

TABLE II

ALIMENTARY

| | | |
|---|---|---|
| Epulis | 1 | |
| Micrognathos and Cleft Palate | 5 | |
| Cleft lip | 4 | |
| Cleft palate | 3 | |
| Cleft lip and palate | 6 | (1 S.B.) |
| Oesophageal atresia | 2 | (1 died) |
| Hiatus Hernia | 1 | |
| Biliary atresia | 2 | |
| Meconium ileus | 1 | (died) |
| Exomphalos | 3 | (1 died, 1 S.B.) |
| Hirschsprung's syndrome | 2 | |
| Imperforate anus | 5 | |
| Persistent cloaca | 1 | (died) |
| TOTAL | 36 | |

Incidence: 0·4 per cent of all births.

avoidance of infection, and some are extremely difficult to feed in the first weeks. The rare occurrence of congenital obstructive lesions of the intestine affords little opportunity for development of surgical skill, but noteworthy are the excellent results that have been obtained by the surgical and anaesthetic team at Southmead hospital in the cases of anal maldevelopment. The one stage "pull-through" operation for imperforate anus saves the babies from months in hospital with colostomies which were always unsatisfactory as the faeces remained fluid, the children malnourished and the urine often infected.

### CONGENITAL HEART DISEASE

Table III shows the incidence of congenital heart disease. In these two years of the review the cyanotic cases all died early but of course a few often survive and some may

TABLE III

CONGENITAL HEART DISEASE
(excluding mongols)

| | | |
|---|---|---|
| Non-cyanotic | 33 | (1 S.B. + 2 died) |
| Cyanotic | 9 | (all died) |
| | 42 | |

Incidence: 0·5 per cent of all births.

RESPIRATORY

| | | |
|---|---|---|
| Diaphragmatic Hernia | 2 | (2 died) |

GENITO-URINARY

| | |
|---|---|
| Hypospadias | 12 |
| Hydrocele | 4 |
| | 16 |

be successfully treated by surgery later. There are quite a large number of non-cyanotic cases and others come to light by the age of three months. The incidence of these cases in neonatal statistics is steadily increasing due to the efficiency of paediatric diagnosis in the newborn. Some of these babies are only first diagnosed when they develop signs of right heart failure during the third or fourth week and many can now be saved by skilled paediatric care at this age who frequently died in the past.

### CENTRAL NERVOUS SYSTEM

The abnormalities of the central nervous system may perhaps be considered of the greatest interest and importance at present because of the considerable advances in treatment recently. Paediatricians in major centres everywhere have become increasingly aware of the numbers of pathetic children with spina bifida cystica and associated hydrocephalus, caused by the Arnold-Chiari malformation of the medulla and cerebellum which plugs the foramen magnum and hence causes hydrocephalus by obstruction of the free flow of cerebrospinal fluid. Table IV shows the large number of

TABLE IV

Central Nervous System

| | | |
|---|---|---|
| Hydrocephalus and Sp. Bifida .. | 27 | (8 S.B.) |
| Anencephaly .. .. .. | 13 | (all died) |
| Meningocele .. .. .. | 5 | |
| Hydrocephalus .. .. .. | 3 | |
| Microcephaly .. .. .. | 1 | |
| Other brain abnormalities .. | 2 | |
| Mongols .. .. .. .. | 16 | (6 Congenital Heart 2 died) |
| Congenital Deafness .. .. | 1 | |
| Congenital Cataracts .. .. | 1 | |
| Total .. .. .. | 69 | |

Incidence: 28 per cent of all malformations.
0·75 per cent of all births.

such cases who were born alive in Southmead Hospital. These children are often regarded as revolting by their parents although some even when untreated are of normal intelligence. They tend to be abandoned in institutions and hence become deprived of parental affection and the stimulus of normal home and community life. The treatment of such children is now being undertaken enthusiastically by such pioneers as Ellison Nash in London, and paediatric and neurosurgeons in some of the northern teaching hospital centres.

The meningomyelocele occurs at any level of the spine or even extends into the occipital bone, but the common site is the lumbosacral region. In this region flaccid paralysis of the legs and neurogenic sphincter disturbances are major associated problems. The rehabilitation programme requires most of the band, and starts with treatment of the meningocele. From the time of birth this must be protected to prevent rupture of the sac and infection. A plastic nylon gravy strainer which can be sterilized daily by boiling is most efficient and is available in several sizes. It is important that no dressings are allowed to touch or press on the sac. Some surgeons prefer to treat this condition as an emergency and operate as quickly as possible after birth, as they claim that there is less risk of infection then or of adhesion of the spinal nerve roots to the sac. Others consider that operation is better performed at three months. In support of the latter view, where there is little fluid or pressure in the sac the membrane frequently becomes epithelialised by this age and operation is sometimes unnecessary. In other cases unfortunately, delay leads to ascending meningitis from which in these days the children often recover but with very severe brain damage or obstructive hydrocephalus. A number of these children inevitably develop progressive hydrocephalus so that this must be tackled before the ventricles enlarge so much that the immature brain becomes permanently damaged or the vision deteriorates. Each case must be judged on its own merits by careful watching of the skull growth, the C.S.F. pressure and the optic fundi. In some cases drainage of C.S.F. from the right lateral ventricle into the blood stream via a special plastic valve inserted into the superior vena cava is carried out almost at once, in others this procedure is delayed until it is clear that the hydrocephalus is progressive. On the whole the results of these procedures, although there are many technical difficulties still to be overcome, justify optimism as so many of the surviving children are able to remain in the normal community after rehabilitation.

At the same time as treatment for the nervous lesion is being carried out, the flaccid legs must receive physiotherapy and orthopaedic treatment to prevent contractures, and encourage residual muscle power, so that the children can eventually walk with calipers. The sphincter disturbances are of perhaps greater importance to life. The anal sphincter can be trained to continence by regular evacuation of the bowel with the use of aperients and suppositories; constipation with secondary megacolon is the problem to be avoided. The neurogenic bladder readily becomes infected so that regular emptying by compression, and free drainage if necessary by transurethral resection of the bladder neck before the age of one year is often needed. The subsequent total incontinence in girls may be remedied by the ileal bladder operation. If this is performed at school age, it is easily managed and compatible with full normal life at school, games and later employment.

A small beginning has been made with this work in Bristol and of course there are teething troubles; we have some failures but there are successes which have proved stimulating and rewarding to those concerned.

TABLE V

SKELETAL

| | | |
|---|---|---|
| Malformations of upper extremity | 9 | (3 absent radius) |
| Malformations of feet and hands | 9 | |
| Polydactyly | 9 | |
| Syndactyly | 4 | |
| Arachnodactyly | 1 | |
| Congenital dysplasia of hip joint | 4 | |
| Craniostenosis | 2 | (1 S.B.) |
| Achondroplasia | 1 | |
| | 39 | |

Incidence: 0·4 per cent of all births.

### PREVENTION OF MALFORMATIONS

Prevention is almost synonymous with paediatrics today, and this may well be the most important aspect of this problem for the future. This would seem to be the essential rôle of the general practitioner and probably one which only he can play. Prevention is difficult without more accurate knowledge of causation but during recent years some exciting new discoveries have been made which have considerable bearing on this problem.

Congenital malformations arise during the first three months of development at the organogenetic stage of the embryo. In the first 18 days of embryonic development implantation and formation of the blastula are dependent mainly on correct environment and radical changes in this probably lead to death of the embryo and abortion. From 18 to 40 days is the really critical time for harmful teratogenic influences. In the 3rd week the primitive streak, notochord and neural folds appear, and in the 4th week, the somites, neural tube, head and tail folds and circulation. During the second month the limb buds and the rudiments of all the organs are formed. During this period, genetic influences are powerful and new knowledge is throwing light on the structure of chromosomes which carry the genes and on the biochemistry of the cell nucleus including the genes. Faulty structure of the twenty-two pairs of somatic chromosomes has been associated with a few cases of multiple abnormality and in particular mongolism; abnormalities of the pattern have sometimes been detected in the mothers too. Abnormalities of the pair of sex chromosomes have been found in some syndromes involving aberrations of genital development. Of even greater importance might be the biochemical discoveries that now reveal the chemical pattern of the gene; i.e. the production from desoxyribonucleic acid, of the 4 amino-acids involved and the enzyme reaction necessary for the normal chemical reactions. Absence of this enzyme formation is responsible for some of the biochemical anomalies, e.g. galactosaemia.

The part played by genes and that played by environmental influences is difficult to determine since both may act together or separately, but the vital fact is the timing. It will be obvious that these serious malformations are determined just after the mother has missed her first menstrual period, when she may be subject to mood changes, fear of pregnancy, general malaise, depression and early pregnancy vomiting. At this stage she may take drugs to relieve her symptoms, and some of these may be teratogenic. Unaware that she might be pregnant, she may be subjected to medical investigation with X-rays or drugs. Many of us have been shocked recently by the reports in the journals of epidemics of malformations of the limbs in infants of mothers who have taken the drug, thalidomide, which has been widely used as an effective and relatively non-toxic sedative.

In recent months we have seen a slight increase in limb malformations, and in one pair of twins with phocomelia of all four limbs, the mother had taken stelazine mg2 b.d. during the first 6 months of pregnancy. Of great importance is the record in recent literature of a pair of non-identical twins with similar limb malformations, indicating that the cause was environmental rather than genetic. It therefore behoves everyone treating a young woman to determine that she is not pregnant before submitting her to investigation or therapy which might be teratogenic. Study of the first three months of pregnancy could be extremely rewarding and its importance has been recognized in Warsaw and Moscow, where education of adolescent girls in preparation for motherhood is being stressed, and women are actively encouraged to report to an obstetric clinic as soon as the first menstrual period has been missed. In this country great strides have been made and emphasis placed on obstetric care during the last three months of pregnancy, but the future may show that study of the first three months is more important and in this the family doctor must play a leading part.

Warkany (1947) wrote: "Teratology, the science of congenital malformations, has until recently been a neglected field in Pediatrics." During the past fifteen years this situation has entirely changed and it can now be said that the subject of congenital malformations is the most important sphere of organic medicine for the paediatrician today.

### REFERENCES

1. Butler, N. (1961); *Brit. M. J.* **1**, 1313.
2. Warkany, J. (1947); Advances in Pediatrics, **2**, Interscience Publishers, New York.

